# Extended Clinical Follow-Up and Long-Term Stuttering Outcomes: A 24-Month Observational Study

**DOI:** 10.3390/healthcare14162576

**Published:** 2026-08-17

**Authors:** Abdulaziz Almudhi

**Affiliations:** Department of Medical Rehabilitation Sciences, College of Applied Medical Sciences, King Khalid University, Abha 61421, Saudi Arabia; almudhi@kku.edu.sa

**Keywords:** stuttering, relapse, longitudinal, structured treatment, therapy

## Abstract

**Background:** Relapse following successful stuttering treatment is a common clinical challenge, yet strategies to support long-term stability after therapy termination remain insufficiently examined. We examined whether sustained clinical follow-up, without additional therapy, was associated with maintenance of treatment gains and a low incidence of relapse in adults who stutter after completion of therapy. **Methods:** In this prospective longitudinal study, 40 adults who stutter completed structured treatment and entered post-treatment follow-up. Nineteen completed 24 months of monthly monitoring, involving a monthly self-rated five-point Speech Satisfaction Rating Scale (SSRS) to track perceived speech satisfaction and to detect relapse, defined a priori as a sustained decline in satisfaction relative to the post-treatment rating. No additional therapy was provided during follow-up. **Results:** Across the 24-month follow-up period, one participant (5.3%) met the predefined criterion for sustained relapse; six participants (32%) showed a transient two-point decline in satisfaction at one or more follow-up points, which had resolved by 24 months in all but one. Speech satisfaction increased significantly from pre-treatment to post-treatment (*p* < 0.001) and remained significantly higher at 24 months compared with pre-treatment levels (*p* < 0.001). Although a modest reduction in speech satisfaction was observed at 12 months (*p* = 0.048), no significant difference was found between post-treatment and 24-month follow-up scores (*p* = 0.109). Longitudinal analysis demonstrated a significant effect of time on speech satisfaction (*p* < 0.001), with no significant effects of age or gender. **Conclusions:** Sustained clinical follow-up without additional therapy was associated with long-term maintenance of self-rated speech satisfaction and a low incidence of sustained relapse over 24 months. These findings suggest that structured follow-up may be a useful component of relapse monitoring in stuttering management, although the single-group design and reliance on a single self-report measure limit causal interpretation.

## 1. Introduction

Stuttering is a neurodevelopmental fluency disorder characterised by disruptions in the normal flow and timing of speech, including sound or syllable repetitions, prolongations, silent or audible blocks, and associated struggle behaviours, which may affect communication confidence and participation. Although some individuals recover during childhood, stuttering may persist into adolescence and adulthood, particularly when symptoms continue beyond the developmental period [1,2]. Therefore, children and adolescents who stutter require treatment approaches that are not only effective in the short and medium term but also support the long-term maintenance of fluency and communication gains.

Establishing what is currently known about recovery and its measurement is a necessary first step, because how recovery is defined and assessed directly shapes how relapse after discharge can later be identified. Several studies highlighted the recovery rate of stuttering therapies. According to [3], the recovery rate for children who stutter ranges from as low as 6.3% to as high as 94%, with a mean recovery rate of 58.7%. Similarly, ref. [4] reported that 39.13% of participants experienced a change in recovery status when reassessed in adolescence and young adulthood, highlighting that recovery is fluid and may continue beyond early childhood. When self-identification was included, recovery classification shifted considerably, with some individuals demonstrating minimal stuttering despite not identifying as recovered, and others self-identifying as recovered despite mild observable disfluencies. Studies examining therapeutic interventions further underscore this variability. Ref. [5] found that adults receiving Multidimensional Individualized Stuttering Therapy (MIST) showed sustained positive outcomes at 24 months, with reductions in stuttering severity maintained rather than traditional recovery rates reported. In contrast, the feasibility trial of Palin Stammering Therapy for School Children aged 8–14 years, Palin STSC (8–14), did not measure recovery directly but demonstrated strong treatment adherence and acceptable outcomes, suggesting potential for future recovery-focused research [6]. Meta-analytic data by [7] reinforce these patterns, showing that recovery likelihood is influenced by factors such as age at onset, sex, language skills, and stuttering severity, with persistent stuttering more likely among males and children with later onset or greater severity.

However, even after recovery, the major challenge faced by the patients is relapse. While initial treatment may yield positive outcomes, stuttering symptoms may re-emerge after several months or even years following the completion of therapy. Several studies highlighted that relapse is often linked to the absence of long-term follow-up care, which is essential to maintain recovery achieved from the therapy. For example, Ref. [8] analyzed 24 adults, showing that factors such as pre-treatment stuttering severity, therapy-related factors, and personality traits significantly influenced relapse. Symptoms may recur in people who stutter unless continuous monitoring or follow-up interventions are provided. This finding highlights the urgent need for long-term clinical follow-up, which aims to track and prevent relapse, thereby protecting the sustainability of treatment effects over the long term. However, this and related work have focused largely on identifying factors that predict relapse risk, rather than on prospectively testing structured, monitoring-only follow-up as the variable of interest; this distinction is the specific gap the present study addresses. As evidenced by [9] therapies effectively reduced stuttering severity and anxiety in participants, and these gains were maintained in the long term.

As previous studies have demonstrated the effectiveness of stuttering treatments in producing positive changes in both internal symptoms, such as psychosocial impact, and external symptoms, such as stuttering severity. However, while treatment effects have been shown to remain stable in the short and medium term, there is still limited research evaluating the long-term maintenance of treatment gains after formal therapy has ended, particularly using structured monitoring designs that track outcomes prospectively across multiple post-discharge time points rather than at a single follow-up assessment. This gap is important because relapse may occur gradually over several months or years and may not be detected if clients are discharged without structured monitoring. It is also recognised that fluency, as indexed by stuttering severity, represents only one dimension of therapeutic success; communicative confidence, self-efficacy, and quality of life are increasingly emphasised within stuttering-acceptance and neurodiversity-affirming frameworks [10]. The present study deliberately bounds its scope to observable speech behaviour and self-rated speech satisfaction as one component of long-term outcome, rather than as a complete account of successful adaptation to stuttering. Therefore, the present study focused on sustained clinical follow-up as a low-intensity monitoring approach rather than as booster therapy. The follow-up sessions were designed to assess self-rated speech satisfaction and to identify early signs of relapse using a predefined criterion, without providing additional therapeutic instruction or fluency training. To reduce the possible clinician effect, all follow-up sessions followed a standardised assessment protocol using the same outcome measures across all time points. The aim of the present study was to describe whether sustained clinical follow-up after stuttering treatment was associated with the long-term maintenance of treatment gains and a low incidence of sustained relapse. Specifically, this study seeks to answer the following questions.

1. Do individuals who stutter remain free of relapse across 24 months of sustained clinical follow-up after treatment?
2. How does speech satisfaction change from pre-treatment to post-treatment and across the 24-month follow-up period as measured by the Five-Point Speech Satisfaction Rating Scale (SSRS)?
3. Is there a statistically significant difference in speech satisfaction between pre-treatment, post-treatment, and the 24-month follow-up?

## 2. Methodology

### 2.1. Study Design

This study employed a prospective observational longitudinal cohort design. A concurrent control or comparison group was not included because withholding structured clinical monitoring from individuals who had completed active stuttering treatment was considered ethically inappropriate, given the clinical importance of early identification of relapse. Accordingly, the study was designed to describe changes in self-rated speech satisfaction during structured follow-up monitoring rather than to establish causal relationships. Outcome measures were assessed at 26 follow-up time points, with each participant serving as their own control by using post-treatment scores as the reference baseline for longitudinal comparisons. The study protocol, including the rationale for the within-participant design, was reviewed and approved by the Research Ethics Committee of King Khalid University (Approval No. KKU-179-2025-31).

Participants were recruited from current clinical cases at the clinic. Initially, 40 adults who stutter were recruited and entered the structured treatment programme. After achieving treatment goals, they transitioned to the structured follow-up phase. During the 24-month follow-up period, several participants discontinued attendance because of relocation, personal commitments, or inability to maintain monthly visits; therefore, they were not included in the final analysis. The final cohort consisted of 19 adults who completed all 24 monthly follow-up sessions. No participant received additional speech–language therapy, psychological intervention, or alternative fluency treatment during the follow-up period. To support consistency across speech samples, all assessments were conducted using the same clinical setting, outcome measures, and assessment protocol. For female participants, appointments were arranged with consideration of participant comfort and availability; however, menstrual-cycle phase was not formally measured or included as an analytic variable.

To ensure consistency across assessments, the same speech–language therapist who provided treatment also conducted the follow-up evaluations. Follow-up sessions were restricted to standardized assessment procedures and did not include therapeutic instruction, corrective feedback, fluency-shaping practice, psychological intervention, or booster therapy. At each session, participants completed SSRS, a brief self-report measure of perceived speech satisfaction, administered under consistent conditions and following the same protocol across all time points to maximize procedural consistency. The study did not include an objective or clinician-rated measure of stuttering severity; self-rated speech satisfaction was the only outcome collected during the follow-up period, and this single-measure design is acknowledged as a limitation. Because the SSRS is completed by the participant, clinician scoring and inter-rater reliability were not applicable.

The speech–language therapist who participated in this research possessed considerable clinical experience in stuttering management and had undergone advanced-level professional training in contemporary therapeutic practices applicable to fluency disorders. The same therapist was involved in the initial treatment phase and also conducted the follow-up assessments. However, the follow-up phase did not include any therapeutic intervention. Instead, the SSRS was completed at every session to monitor long-term perceived speech satisfaction and to identify possible relapse patterns. To reduce the potential clinician effect, follow-up visits were limited to assessment and monitoring only, with no therapeutic instruction, corrective feedback, fluency shaping practice, psychological intervention, or booster therapy provided during the follow-up period.

### 2.2. Participants

The characteristics of the individuals who took part in the different stages of the study, baseline, post-treatment, and the final 24-month follow-up, are summarized in Section 3. Inclusion criteria required that participants (a) were adults seeking treatment for developmental stuttering, (b) demonstrated clear and clinically verified instances of stuttering in a recorded speech sample, and (c) were able to commit to regular therapy sessions followed by monthly monitoring. Exclusion criteria included (a) a diagnosed neurological condition, (b) additional communication disorders or co-occurring difficulties that could influence fluency, and (c) involvement in any other speech–language or psychological intervention during the follow-up period. Forty adults initially entered the treatment programme and completed both the baseline and post-treatment assessments. The final sample consisted of 19 adults who completed all 24 monthly follow-up assessments. Attrition occurred for several reasons, most commonly relocation or personal circumstances that made it difficult to maintain regular attendance. All participants provided written informed consent before entering the study.

### 2.3. Clinical Treatment Phase

Each participant received a rigorous and individualised protocol of stuttering treatment before moving into the follow-up phase. This early phase of treatment aimed to enable each client to have greater control over speech fluency, fewer behaviours associated with physical struggle, and more practical strategies to manage speaking situations with confidence. The treatment approach included fluency-management and stuttering-modification techniques, such as controlled speech rate, easy onset, smooth speech production, pausing, breathing control, reduction in physical tension, and strategies for managing moments of stuttering during conversation. Therapy also focused on transferring these strategies from the clinical setting to real-life communication situations.

Treatment success was determined using a combination of behavioural observations, client self-reports, recorded conversational speech samples, and stability of clinical ratings across consecutive sessions (see Table 1). Participants were moved from the active therapy phase to the follow-up phase when they showed steady improvement in observable stuttering behaviours, greater ease of communication, reduced physical struggle, improved speech satisfaction, and stable clinical ratings over repeated sessions. Although these indicators usually marked the termination of formal therapy, clients were not discharged immediately. Instead, they were entered into a structured monthly follow-up phase because relapse after fluency gains may emerge gradually several months after therapy has stopped. Follow-up was therefore continued as a systematic monitoring process to check for early regression and to identify any gradual re-emergence of stuttering behaviours that might not be detected immediately after treatment. No further therapeutic instruction, corrective feedback, fluency training, psychological intervention, or booster therapy was provided during this phase. As shown in Table 1, mean SSRS scores increased from 1.21 before treatment to 4.42 after treatment and remained elevated at 24 months, with a mean score of 4.11. This pattern indicates sustained improvement in perceived speech satisfaction across the follow-up period.

### 2.4. Follow-Up Protocol

#### 2.4.1. Stage 1 (Post-Treatment Entry)

Participants who completed their individualised stuttering therapy and demonstrated stable improvements were invited to participate in the follow-up phase. Written consent was obtained at this stage. During the first follow-up visit, each client completed the SSRS. This patient-reported rating was included because stuttering outcomes should capture the speaker’s own communication experience [10]. All assessments were conducted in the clinician’s therapy room under consistent environmental conditions. The same speech–language therapist conducted all interactions to ensure uniformity across samples. This assessment served as the baseline reference for the follow-up period.

#### 2.4.2. Stage 2 (Monthly Follow-Up Sessions)

The participants had one follow-up appointment after every 1 month in the course of 24 months. At each session, participants completed the SSRS to provide a monthly rating of their satisfaction with their speech. For female participants, appointments were scheduled, where feasible, outside the active menstrual phase to reduce possible variability in speech and voice sampling, as previous studies have reported menstrual-cycle-related changes in laryngeal function, vocal quality, pitch, intensity, and self-perceived vocal symptoms [11,12,13]. There was no reception of a therapeutic intervention in all these sessions, but it was mainly about monitoring perceived speech satisfaction, recording changes, and detecting the initial symptoms of potential relapse. Each of the assessments was conducted by the same clinician to ensure consistency in administration and interaction style.

#### 2.4.3. Stage 3 (Final 24-Month Assessment)

The final assessment session was carried out at the end of the 24 months’ follow-up period on clients who stayed in the study. The participants again rated their speech satisfaction using the SSRS. The comparison of these endpoint measures with initial post-treatment results was done in order to assess long-term maintenance of treatment gains. Those participants who dropped out of attending or could not finish all the follow-up sessions were not included in the final analysis.

### 2.5. Data Collection

Participants completed an individualized therapy programme before entering the structured follow-up phase. Following treatment completion, clients attended an initial post-therapy assessment session during which the SSRS was administered. The SSRS provides a subjective rating of perceived speech satisfaction, anchored from 1 = very dissatisfied to 5 = very satisfied. This rating formed the baseline score against which all subsequent evaluations were compared. No objective or clinician-rated measure of stuttering severity was collected at any stage of the study.

Following the post-treatment assessment, participants entered a 24-month follow-up protocol consisting of monthly clinical visits. At each visit, a single outcome measure was collected: a self-reported SSRS rating of perceived speech satisfaction. A total of 26 measurement points were therefore obtained for every client (baseline, immediate post-treatment, and 24 consecutive monthly assessments). All sessions were conducted under controlled clinical conditions to minimise variability in acoustic and interactional factors. To further support consistency, all assessments were administered by the same speech–language therapist, following a standardized protocol across all time points.

The SSRS is a brief patient-reported measure of perceived speech satisfaction, scored on a five-point scale ranging from 1 (very dissatisfied) to 5 (very satisfied). The instrument was selected because of its clinical practicality and suitability for repeated monthly administration throughout the follow-up period. The SSRS was used as a clinical rating measure of speech satisfaction and was not intended to function as a comprehensive psychometric instrument assessing broader domains such as communication participation, emotional impact, or quality of life.

Special scheduling considerations were applied for female clients: follow-up appointments were arranged to avoid menstrual-cycle periods, based on clinical observations indicating that hormonal fluctuations sometimes influence comfort during speech sampling. No therapeutic intervention was provided during follow-up visits; the purpose of these sessions was strictly to document perceived speech satisfaction and to detect early indications of relapse. The structured collection of SSRS data over 26 occasions enabled a detailed examination of the long-term stability of self-rated speech satisfaction for individuals who had previously met their therapeutic goals.

### 2.6. Operational Definition of Relapse

For the purposes of this study, relapse was defined using a predetermined quantitative criterion based on the participant’s self-rated speech satisfaction (SSRS) relative to the immediate post-treatment assessment. Because no objective or clinician-rated severity measure was collected, relapse refers specifically to a sustained reduction in self-rated speech satisfaction. A participant was considered to have relapsed if any of the following occurred and was still present at the 24-month endpoint:

1. A decline of two or more points on the SSRS relative to the post-treatment rating that remained present at the 24-month endpoint (i.e., a sustained rather than transient decline).
2. Month-to-month fluctuations that resolved before the 24-month endpoint were recorded but not classified as sustained relapse; the frequency of transient and sustained declines is reported in Section 3.

The relapse criterion was established a priori, before data collection, and was specified within the approved study protocol. A decline of two or more points on the SSRS was considered reflective of a meaningful reduction in self-perceived speech satisfaction. Requiring the decline to be sustained at the 24-month endpoint, rather than counting any single-session dip, was intended to minimise misclassification arising from isolated fluctuations in a single self-report measure.

### 2.7. Data Management and Statistical Analysis

Data from the 26 SSRS assessment points were entered into a spreadsheet containing client identifiers, session numbers, and satisfaction ratings. Prior to analysis, data were screened to ensure completeness and accuracy, and any inconsistency was verified against original clinical records. Statistical computations were performed in IBM SPSS Statistics (Version 27.0).

The analysis provided descriptive statistics (mean, standard deviation, and range) of SSRS ratings at all-time points. The Shapiro–Wilk test was used to assess the normality of the SSRS scores. Repeated-measures ANOVA (in cases of normally distributed data) or the Friedman test (in the case of non-parametric data) was utilized in the longitudinal analysis in order to test the changes over the 24-month follow-up. Where necessary, post hoc pairwise comparisons adjusted by Bonferroni were applied to ascertain variations across time points.

Non-parametric repeated-measures tests were used to investigate the change in perceived speech satisfaction with time, relying on the SSRS. Changes in SSRS post-treatment compared to pre-treatment and changes in SSRS 24 months post-treatment were evaluated with the Friedman test. Post-hoc comparisons were done using a Wilcoxon signed-rank test where the Friedman test indicated a statistically significant effect between the two time points of assessment (pre-treatment vs. post-treatment, post-treatment vs. 24-month follow-up, and pre-treatment vs. 24-month follow-up). To estimate the magnitude of observed changes, effect sizes were computed with Kendall W being reported when Friedman was used and Cohen d when Wilcoxon comparisons were done. Every test was two-tailed, and the level of statistical significance was *p* < 0.05. In the cases where several pairwise comparisons were done, they were adjusted by the necessary measures to address Type I error. All the analyses were carried out in SPSS software (Version 27.0).

## 3. Results

### 3.1. Participant Characteristics

A total of 19 participants were included in the final analysis. The sample included 11 male participants and 8 female participants. All participants completed the post-treatment assessment and all 24 monthly follow-up assessments; therefore, there were no missing data for the variables included in the final longitudinal analysis. Demographic variables were summarised descriptively rather than analysed as continuous outcomes, as shown in Table 2.

### 3.2. Reliability of the Speech Satisfaction Rating Scale

All 19 participants completed the pre-treatment, post-treatment, and 24-month follow-up assessments. The internal consistency of the SSRS across the 26 measurement points was excellent (Cronbach’s α = 0.955), indicating high reliability of the scale over time (see Table 3).

### 3.3. Overall Change in Speech Satisfaction over Time

To examine whether perceived speech satisfaction changed over time, SSRS scores measured at pre-treatment, post-treatment, and monthly follow-ups over 24 months were analysed using a Friedman test. The analysis revealed a statistically significant overall effect of time on SSRS scores, χ^2^ (25) = 106.07, *p* < 0.001, indicating that speech satisfaction differed significantly across assessment points (Table 4).

### 3.4. Pairwise Comparisons of Speech Satisfaction Across Key Time Points

Post-hoc pairwise comparisons were conducted using Wilcoxon signed-rank tests to identify differences between clinically relevant time points (Table 5). The corresponding rank distributions for these comparisons are presented in Appendix A (Table A1). A significant improvement in speech satisfaction was observed from pre-treatment to post-treatment (Z = −3.89, *p* < 0.001).

When post-treatment scores were compared with follow-up assessments, a modest but statistically significant reduction in SSRS scores was observed between post-treatment and 12-month follow-up (Z = −1.98, *p* = 0.048). However, no statistically significant difference was found between post-treatment and 24-month follow-up scores (Z = −1.60, *p* = 0.109), suggesting that post-treatment gains were largely maintained over the long term.

Importantly, SSRS scores at the 24-month follow-up remained significantly higher than pre-treatment scores (Z = −3.88, *p* < 0.001), indicating sustained improvement in perceived speech satisfaction over the 24-month period.

### 3.5. Longitudinal Descriptive Trends by Gender (Optional)

Descriptive statistics for SSRS scores across pre-treatment, post-treatment, and monthly follow-ups stratified by gender are presented in Table 6. At pre-treatment, mean SSRS scores were low for both males (M = 1.27, SD = 0.47) and females (M = 1.13, SD = 0.35). Following treatment, substantial improvements were observed in both groups, with post-treatment mean SSRS scores increasing to 4.27 (SD = 0.79) for males and 4.63 (SD = 0.52) for females. Across the 24-month follow-up period, mean SSRS scores for both genders remained consistently higher than pre-treatment levels, with minor fluctuations observed at individual follow-up points. At the 24-month follow-up, mean SSRS scores remained elevated for both males (M = 3.91, SD = 0.54) and females (M = 4.38, SD = 0.74), indicating sustained improvements in perceived speech satisfaction over time.

### 3.6. Parametric Sensitivity Analysis

As a sensitivity analysis, a repeated-measures ANOVA was conducted to confirm the non-parametric findings. Mauchly’s test of sphericity indicated a violation of the sphericity assumption (W ≈ 0.00); therefore, Greenhouse–Geisser corrections were applied (ε = 0.335). The corrected analysis revealed a significant main effect of time on SSRS scores, F(8.38, 134.03) = 4.38, *p* < 0.001, with a moderate effect size (partial η^2^ = 0.215), supporting the results of the Friedman test. Detailed within-subject effects are provided in Appendix A (Table A2). No significant interactions were observed between time and age, F(8.38, 134.03) = 1.51, *p* = 0.157, partial η^2^ = 0.086, or between time and gender, F(8.38, 134.03) = 1.39, *p* = 0.202, partial η^2^ = 0.080, indicating that changes in speech satisfaction over time were consistent across demographic groups (Table 7).

Relapse was operationalised as a two-point decline in SSRS relative to the post-treatment rating that remained present at the 24-month endpoint (Section 2.6). Six of the 19 participants (32%) recorded a two-point decline in satisfaction at one or more monthly follow-up points; in every instance this was a decline from a post-treatment rating of 5 to a follow-up rating of 3, and no participant fell below the scale midpoint or exceeded a two-point drop at any assessment. In five of these participants, the decline was transient, with satisfaction returning to within one point of the post-treatment rating by 24 months. One participant remained two points below the post-treatment rating at the 24-month endpoint, meeting the criterion for a sustained decline. Sustained relapse by the predefined criterion therefore occurred in one participant (5.3%), whereas transient month-to-month fluctuation that did not persist to the endpoint was more common (six participants). These descriptive findings should be interpreted in light of the single-group design and the use of a single self-report measure.

## 4. Discussion

This study found that positive results were sustained throughout the 24 months when assessed with the repeated use of the Speech Satisfaction Rating Scale. However, a more accurate question of how chronic clinical follow-up helped to produce these results and what specific elements of follow-up were used to protect long-term stability are challenging to answer and, ultimately, are out of the scope of this paper. When examining the longitudinal assessment trends as they are seen among the respondents, however, it seems that the general stability with regard to speech satisfaction is more directly linked to the systematic, periodic presence of follow-up than to any systematic therapeutic intervention. As a result, the discussion which appears below is limited by the description of the factors of the sustained clinical follow-up, thus providing the required background to the hypothesis that continuous monitoring may have contributed to the strong and long-lasting maintenance of post-treatment gains.

The results of the current study provide empirical evidence for the theoretical approaches, which are promoted by [14,15], who promote an individualized approach and context-specific methods of managing stuttering in the long term. In such descriptions, long-term clinical follow-up is conceived as a process with more than one dimension, and there is no simple and linear cause-and-effect correlation between participation in follow-up sessions and the consistency of results at different points of assessment. Instead, the follow-up model is a qualitative change in clinical practice, one where a continued interaction and self-assessment by the stutterer are the focus, and not the perpetual use of treatment methods by the therapist. The overall and long-term results noticed over time indicate that peculiarities of long-term follow-up are the topic that should be investigated further and can be relevant.

### 4.1. Sustained Clinical Follow-Up in the Context of Existing

#### Approaches

The outcomes show that there is a strong improvement in the speech satisfaction level between the pre-treatment baseline and post-treatment evaluation, which has been maintained over a 24-month follow-up period. This stability is remarkable and contrasts with the results provided by researchers in which relapse has been observed after the end of treatment [16,17]. Long-term follow-up evidence is used to show that stuttering outcomes are significantly recovered and stable both during childhood and adolescence. A 14-year follow-up study had a recovery rate of 65.6 percent, and 97 percent of respondents had fewer than 3 percent of syllables stuttered and no relapse between mid-childhood and late adolescence [3]. Psychosocial outcomes also report lasting outcomes, with intensive stuttering therapy correlated with a steady or further minimized degree of stuttering and influenced psychosocial outcomes more than 10 years after treatment [2]. Cognitive-behavioural play therapy produced social anxiety reduction in school-aged children with stuttering, and the success of play therapy was sustained in subsequent evaluation periods [18]. However, follow-up studies over a long-term period on children with co-occurring stuttering and speech-sound disorder showed that the results of both speech and phonology were largely stable, but 2 out of 4 children relapsed [19]. The direct comparison to the results obtained with other interventions for stuttering has been difficult due to the dissimilarity in the outcome measures, time of follow-up, and the terms that are applied to decide a relapse. Nevertheless, only a low incidence of sustained relapse was observed in the current study, which is consistent with the possibility that formal and extended follow-up may help maintain treatment gains even when no additional therapeutic intervention is provided.

There are other properly documented methods of managing stuttering that have recorded good results, especially in those methods involving speech aspects coupled with the use of cognitive or psychosocial elements. Various studies indicated that there are improvements in fluency and communication-related experiences, but the sustainability of the gains in the long-term follow-up is not always uniformly reported. New data also show the significance of working on cognitive, emotional, and attentional parameters as well as speech-centred treatment. Studies of cluttering have revealed that patients have a high rate of presenting with high anxiety, somatic complaints, attention problems, and high alexithymia scores, and reduced emotional awareness has been correlated with low mental wellbeing [20]. Additional results on the non-combinative groups of functional speech disorders show that multidisciplinary speech and language therapy may result in relevant clinical improvement, and most patients exhibited symptomatic improvement after structured intervention [21]. The existence of broader theoretical frameworks, including the Framework of Understanding Effortful Listening, further demonstrates the impact of attentional capacity, motivation, and cognitive energy on the communication requirements, and how cognitive load and emotional regulation contribute to speech-related disorders [22]. Comparatively, the current study shows that the level of speech satisfaction was still high, 12 and 24 months after treatment was completed. This observation indicates that, in the absence of further therapy, clinical follow-up could be adequate to sustain long-term stability in some stuttering individuals, especially in cases where treatment objectives have been well met before follow-up.

As suggested in past studies, an intervention that focuses on fluency shaping, or speech restructuring, can yield significant improvements in the observable stuttering, but perhaps not be effective in treating more general experiential dimensions of communication. This difference between apparent fluency improvement and more general communicative experience can be seen through empirical research of fluency-shaping interventions and speech restructuring to demonstrate how these different systems relate to each other. School-age children have shown significant and long-lasting decreases in stuttered syllables after intensive speech restructuring models based on a group setting, and with a structured maintenance phase, but results on speech naturalness and experiential outcome are less predictable [23]. Additional theory-building on integrated cognitive-behavioural fluency-enhancing interventions also emphasizes the role of motivational, emotional, and effort-based processes, and contextual factors, including family and school support, in the process of determining longer-term treatment outcomes [24]. These results show that although techniques that aim at enhancing fluency can consistently decrease stuttering frequency, the long-term quality of communication can be related to the extent of treatment success in cognitive, emotional, and situational aspects as well as speech mechanics. Though the current research did not directly evaluate psychosocial variables like anxiety or fear of negative assessment, the fact that there was steady improvement in speech satisfaction implies that the respondents could incorporate post-treatment speech into the daily communicative situations in the long run. This study cannot establish whether sustained follow-up is more effective than other therapeutic approaches, nor that it caused the observed maintenance of gains; rather, the findings suggest that structured follow-up is associated with maintained stability and warrants further investigation as a potential element of stuttering management.

## 5. Clinical Implications

Although it is generally accepted in the stuttering therapy sector that long-term outcomes are as significant as short-term fluency improvement, there is no systematic study on how stability of treatment must be maintained after formal intervention. The active therapy technique is the focus of most of the stuttering strategies, and the post-treatment phase is considered secondary or optional. In this cohort, long-term clinical follow-up was associated with prolonged treatment gains and a low incidence of delayed relapse, and the stability of speech satisfaction observed without further therapeutic input raises the possibility that continued clinical contact may play a stabilizing role through monitoring, introspection, and accountability. This view is consistent with the individual-in-context approaches that stress that long-term results do not just rely on the acquisition of techniques, but also on how individuals incorporate speech changes in communicative contexts in the long run.

Notably, its longer-term follow-up focus does not place it as a therapist versus or alternative to the traditional stuttering treatment methods, such as fluency shaping, speech restructuring, or cognitive-behavioural interventions. Instead, follow-up can be considered as a continuation of these interventions, which contributes to their sustainability after the end of the therapy. Similar to the personalized and process-oriented models of treatment, the direct cause–effect relationships between specific elements of follow-up and long-term outcomes are difficult to identify as a result of the complexity and individual variation in stuttering. However, the low incidence of sustained relapse in the given study indicates the possibility of clinical relevance of structured follow-up as a resource-efficient, low-intensity strategy. The same findings lend credence to an abrupt discharge practice needing to be replaced by follow-up-oriented care pathways that acknowledge stuttering as a dynamic condition that needs continuous, although not necessarily intensive, clinical support over time.

## 6. Strengths and Limitations

The current study has an advantage in its long longitudinal design, which allowed systematic monitoring of the steadiness of treatment outcomes over a period of 24 months through repeated and standardised assessments. The use of multiple measurement points per participant gave a fine level of description of long-term change and minimised reliance on individual post-treatment findings, which has been documented as a weakness in the literature on stuttering. Baseline and post-treatment measures played the role of within-participant reference points; however, the lack of a control or comparison group during the follow-up period has to be recognised as a relevant limitation to the interpretation of the results. Therefore, although the findings suggest that extended clinical follow-up was associated with maintenance of gains and a low incidence of sustained relapse, causality cannot be confirmed.

Although much effort was put into ensuring consistency and reliability by using a standardised assessment protocol and the participation of one experienced clinician, the effects of extraneous variables during the 24-month follow-up period cannot be completely disregarded. The same clinician was involved in treatment and follow-up assessment, which supported consistency in scoring and interaction style, but may also have introduced a possible clinician effect or observer bias. In addition, the absence of an independent blinded assessor should be recognised as a methodological limitation. In addition, the study is limited by its reliance on a small final sample, which can be explained by the loss of participants during the follow-up period, and this may limit the applicability and generalisability of the findings.

Another weakness is related to the absence of direct measurements of psychosocial variables such as anxiety, avoidance, fear of negative evaluation, communication participation, or quality of life, which limits understanding of the processes involved in maintaining satisfaction with speech over a long time. Although the Speech Satisfaction Rating Scale provides clinically applicable data about the lived experience of communication of participants, it was used as a brief patient-reported clinical rating rather than as a fully standardised multidimensional psychometric instrument. Therefore, incorporation of more validated psychosocial and quality-of-life indicators would have made the results more interpretative. Relatedly, the study relied on a single self-reported satisfaction measure and did not include any objective or clinician-rated index of stuttering severity, which limits the scope of the findings; this reliance should not be read as treating fluency as the only or primary marker of successful outcome, stuttering-acceptance and neurodiversity-affirming frameworks emphasise that communicative confidence and identity-related outcomes can diverge from fluency metrics, and future work should pair behavioural measures such as the SSI-4 with acceptance-based outcome measures to capture this dimension.

A further limitation concerns the scheduling of female participants outside the active menstrual phase, where feasible. This approach was used to reduce possible variability in repeated speech and voice recordings; however, menstrual-cycle phase was not formally analysed as an independent variable. Therefore, its possible association with stuttering severity, speech satisfaction, or relapse-related outcomes could not be examined in the present study. Future studies should prospectively record menstrual-cycle phase and analyse it as a variable of interest rather than relying only on procedural scheduling.

It is also possible that self-selection bias had its effect; the participants who remained involved in the long-term follow-up were probably highly motivated and able to invest in consistent clinical follow-ups. In addition, participant attrition during the follow-up period may have influenced the composition of the final sample, and the possibility of attrition bias cannot be excluded. The results, therefore, might not be applicable to all individuals who stutter, especially those who show less consistent treatment responses or have limited access to continued care. However, the results indicate the experiences of a clinically relevant group of adults who stutter and highlight the possible worth of continued follow-up as a part of routine stuttering management. Further studies that include a bigger sample, comparison group, independent blinded assessors, multidimensional outcome measures, and formal analysis of participant-related variables would assist in a more detailed clarification of the role and mechanisms linked with extended clinical follow-up in relapse prevention.

## 7. Conclusions

In this single-group cohort, sustained clinical follow-up without additional therapy was associated with maintenance of treatment gains and a low incidence of sustained relapse over 24 months, and self-rated speech satisfaction remained largely stable across that period. Because no comparison group was included, these findings cannot establish that follow-up itself caused this stability; natural maintenance among clients who had already met rigorous discharge criteria, and self-selection among the highly motivated participants who remained in the study remain plausible alternative explanations. The findings should therefore be regarded as hypothesis-generating rather than confirmatory. They suggest that structured post-discharge monitoring is worth investigating further as a potential, low-intensity component of stuttering management, using controlled designs, comparison groups, and outcome measures that extend beyond fluency to capture communicative confidence and quality of life.

## Figures and Tables

**Table 1:** Changes in Speech Satisfaction Following Stuttering Treatment.

Assessment Point	*n*	Mean SSRS Score	SD	Range	Interpretation
Pre-treatment	19	1.21	0.42	1–2	Low speech satisfaction before treatment
Post-treatment	19	4.42	0.69	3–5	Marked improvement after treatment
12-month follow-up	19	3.95	0.71	3–5	Slight reduction but scores remained high
24-month follow-up	19	4.11	0.66	3–5	Treatment gains largely maintained

**Table 2:** Demographic Characteristics of Participants Included.

Characteristic	*n*	%
Male	11	57.9
Female	8	42.1
Total sample	19	100.0
Completed 24-month follow-up	19	100.0

**Table 3:** Reliability Statistics of the Speech Satisfaction Rating Scale (SSRS).

Reliability Statistics	
Cronbach’s Alpha	*n* of Items
0.955	26

**Table 4:** Friedman Test Statistics for Changes in Speech Satisfaction Scores Over Time.

Test Statistics ^a^	
*n*	19
Chi-Square	106.067
df	25
Asymp. Sig.	0.000

a. Friedman Test.

**Table 5:** Pairwise Comparison of Speech Satisfaction Scores Between Key Assessment Time Points Using Wilcoxon Signed-Rank Test.

Test Statistics ^a^				
	SSRS-POST-SSRS-PRE	SSRS-FU12-SSRS-POST	SSRS-FU24-SSRS-POST	SSRS-FU24-SSRS-PRE
Z	−3.886 ^b^	−1.979 ^c^	−1.604 ^c^	−3.880 ^b^
Asymp. Sig. (2-tailed)	0.000	0.048	0.109	0.000

a. Wilcoxon Signed Ranks Test. b. Based on negative ranks. c. Based on positive ranks.

**Table 6:** Descriptive Statistics of SSRS Scores by Gender Over 24-Month Follow-Up.

Descriptive Statistics				
	Gender	Mean	Std. Deviation	*n*
SSRS-PRE	Male	1.27	0.467	11
	Female	1.13	0.354	8
	Total	1.21	0.419	19
SSRS-POST	Male	4.27	0.786	11
	Female	4.63	0.518	8
	Total	4.42	0.692	19
SSRS-FU1	Male	4.00	0.775	11
	Female	4.63	0.744	8
	Total	4.26	0.806	19
SSRS-FU2	Male	4.09	0.944	11
	Female	4.50	0.535	8
	Total	4.26	0.806	19
SSRS-FU3	Male	4.00	0.775	11
	Female	4.63	0.518	8
	Total	4.26	0.733	19
SSRS-FU4	Male	4.27	0.786	11
	Female	4.38	0.744	8
	Total	4.32	0.749	19
SSRS-FU5	Male	4.09	0.831	11
	Female	4.63	0.744	8
	Total	4.32	0.820	19
SSRS-FU6	Male	4.09	0.701	11
	Female	4.50	0.756	8
	Total	4.26	0.733	19
SSRS-FU7	Male	3.73	0.647	11
	Female	4.75	0.463	8
	Total	4.16	0.765	19
SSRS-FU8	Male	3.73	0.647	11
	Female	4.63	0.518	8
	Total	4.11	0.737	19
SSRS-FU9	Male	3.64	0.674	11
	Female	4.63	0.744	8
	Total	4.05	0.848	19
SSRS-FU10	Male	3.91	0.539	11
	Female	4.50	0.535	8
	Total	4.16	0.602	19
SSRS-FU11	Male	3.82	0.603	11
	Female	4.75	0.463	8
	Total	4.21	0.713	19
SSRS-FU12	Male	3.64	0.674	11
	Female	4.38	0.518	8
	Total	3.95	0.705	19
SSRS-FU13	Male	3.64	0.674	11
	Female	4.75	0.463	8
	Total	4.11	0.809	19
SSRS-FU14	Male	3.64	0.674	11
	Female	4.38	0.518	8
	Total	3.95	0.705	19
SSRS-FU15	Male	3.82	0.751	11
	Female	4.25	0.707	8
	Total	4.00	0.745	19
SSRS-FU16	Male	3.82	0.603	11
	Female	4.13	0.641	8
	Total	3.95	0.621	19
SSRS-FU17	Male	3.82	0.603	11
	Female	4.50	0.756	8
	Total	4.11	0.737	19
SSRS-FU18	Male	3.82	0.603	11
	Female	4.25	0.886	8
	Total	4.00	0.745	19
SSRS-FU19	Male	3.82	0.405	11
	Female	4.50	0.756	8
	Total	4.11	0.658	19
SSRS-FU20	Male	4.00	0.447	11
	Female	4.38	0.744	8
	Total	4.16	0.602	19
SSRS-FU21	Male	3.91	0.539	11
	Female	4.50	0.535	8
	Total	4.16	0.602	19
SSRS-FU22	Male	3.91	0.701	11
	Female	4.38	0.744	8
	Total	4.11	0.737	19
SSRS-FU23	Male	4.09	0.539	11
	Female	4.63	0.518	8
	Total	4.32	0.582	19
SSRS-FU24	Male	3.91	0.539	11
	Female	4.38	0.744	8
	Total	4.11	0.658	19

**Table 7:** Mauchly’s Test of Sphericity for Longitudinal SSRS Analysis.

Mauchly’s Test of Sphericity ^a^							
Measure: MEASURE_1							
Within-Subjects Effect	Mauchly’s W	Approx. Chi-Square	df	Sig.	Epsilon ^b^		
					Greenhouse–Geisser	Huynh–Feldt	Lower-Bound
factor1	0.000	.	324	.	0.335	0.825	0.040
Tests the null hypothesis that the error covariance matrix of the orthonormalized transformed dependent variables is proportional to an identity matrix.							

a. Design: Intercept + AGE + GENDER. Within-Subjects Design: factor1. b. May be used to adjust the degrees of freedom for the averaged tests of significance. Corrected tests are displayed in the Tests of Within-Subjects Effects table.

## Data Availability

The data supporting the findings of this study are available from the corresponding author upon reasonable request. This dataset contains clinical follow-up information collected from human participants over 24 months, including repeated assessments and highly sensitive speech-related data. Due to ethical restrictions, participant confidentiality, and the terms approved by the Research Ethics Committee, the full dataset cannot be publicly shared. However, anonymized data can be provided upon reasonable request to qualified researchers, in compliance with ethical guidelines and participant privacy protections.

## Appendix A

**Table A1:** Wilcoxon Signed-Rank Test Ranks for SSRS Score Comparisons.

Ranks				
		*n*	Mean Rank	Sum of Ranks
SSRS-POST-SSRS-PRE	Negative Ranks	0 ^a^	0.00	0.00
	Positive Ranks	19 ^b^	10.00	190.00
	Ties	0 ^c^		
	Total	19		
SSRS-FU12-SSRS-POST	Negative Ranks	9 ^d^	7.00	63.00
	Positive Ranks	3 ^e^	5.00	15.00
	Ties	7 ^f^		
	Total	19		
SSRS-FU24-SSRS-POST	Negative Ranks	8 ^g^	6.19	49.50
	Positive Ranks	3 ^h^	5.50	16.50
	Ties	8 ^i^		
	Total	19		
SSRS-FU24-SSRS-PRE	Negative Ranks	0 ^j^	0.00	0.00
	Positive Ranks	19 ^k^	10.00	190.00
	Ties	0 ^l^		
	**Total**	19		

a. SSRS-POST < SSRS-PRE. b. SSRS-POST > SSRS-PRE. c. SSRS-POST = SSRS-PRE. d. SSRS-FU12 < SSRS-POST. e. SSRS-FU12 > SSRS-POST. f. SSRS-FU12 = SSRS-POST. g. SSRS-FU24 < SSRS-POST. h. SSRS-FU24 > SSRS-POST. i. SSRS-FU24 = SSRS-POST. j. SSRS-FU24 < SSRS-PRE. k. SSRS-FU24 > SSRS-PRE. l. SSRS-FU24 = SSRS-PRE.

**Table A2:** Tests of Within-Subjects Effects for Longitudinal SSRS Analysis.

Tests of Within-Subjects Effects							
Measure: MEASURE_1							
Source		Type III Sum of Squares	df	Mean Square	F	Sig.	Partial Eta Squared
factor1	Sphericity Assumed	29.042	25	1.162	4.378	0.000	0.215
	Greenhouse–Geisser	29.042	8.377	3.467	4.378	0.000	0.215
	Huynh–Feldt	29.042	20.615	1.409	4.378	0.000	0.215
	Lower-bound	29.042	1.000	29.042	4.378	0.053	0.215
factor1 * AGE	Sphericity Assumed	9.988	25	0.400	1.506	0.058	0.086
	Greenhouse–Geisser	9.988	8.377	1.192	1.506	0.157	0.086
	Huynh–Feldt	9.988	20.615	0.485	1.506	0.074	0.086
	Lower-bound	9.988	1.000	9.988	1.506	0.238	0.086
factor1 * GENDER	Sphericity Assumed	9.242	25	0.370	1.393	0.101	0.080
	Greenhouse–Geisser	9.242	8.377	1.103	1.393	0.202	0.080
	Huynh–Feldt	9.242	20.615	0.448	1.393	0.120	0.080
	Lower-bound	9.242	1.000	9.242	1.393	0.255	0.080
Error(factor1)	Sphericity Assumed	106.124	400	0.265			
	Greenhouse–Geisser	106.124	134.025	0.792			
	Huynh–Feldt	106.124	329.833	0.322			
	Lower-bound	106.124	16.000	6.633			

* indicates interaction effects between variables (factor1 × AGE and factor1 × GENDER).
